# Use of the Corus® CAD Gene Expression Test for Assessment of Obstructive Coronary Artery Disease Likelihood in Symptomatic Non-Diabetic Patients

**DOI:** 10.1371/currents.eogt.0f04f6081905998fa92b99593478aeab

**Published:** 2013-08-26

**Authors:** Jose Vargas, Joao A.C. Lima, William E. Kraus, Pamela S Douglas, Steven Rosenberg

**Affiliations:** MedStar Georgetown University Hospital, Washington, DC, USA; Division of Cardiology, Johns Hopkins Hospital, Baltimore, Maryland, USA; School of Medicine, Duke University, Durham, North Carolina, USA; Duke Clinical Research, Duke University, Durham, North Carolina, USA; CardioDx, Inc., Palo Alto, California, USA

## Abstract

The determination of the underlying etiology of symptoms suggestive of obstructive coronary artery disease (CAD, ≥50% stenosis in a major coronary artery) is a common clinical challenge in both primary care and cardiology clinics. Usual care in low to medium risk patients often involves a family history, risk factor assessment, and then stress testing with or without non-invasive imaging. If positive, this is often followed by invasive coronary angiography (ICA). Despite extensive adoption of this usual care paradigm, more than 60% of patients referred for angiography do not have obstructive CAD. In order to robustly identify those symptomatic patients without obstructive CAD, who can avoid subsequent cardiac testing and look elsewhere for the cause of their symptoms, a recently described whole blood gene expression score (GES: Corus® CAD, CardioDx, Inc., Palo Alto, CA) has been developed and validated in two multi-center trials. This paper reviews the published literature and assessments by independent parties regarding the analytical and clinical validity as well as the clinical utility of the Corus® CAD test.

## Clinical Scenarios

Coronary artery disease (CAD) and its clinical sequelae, including myocardial infarction and heart failure, are the leading causes of morbidity and mortality in the developed and developing world. Symptoms consistent with CAD are common, variable and quite diverse with significant gender-specific differences and overlap with other common conditions. Symptomatic patients are often first seen by primary care physicians who determine if a referral to a cardiology service is warranted, prior to investigation of other causes for the symptoms. In cardiology practices, current practice guidelines suggest non-invasive imaging for medium risk patients and invasive coronary angiography for high risk patients 1. Despite the utilization of non-invasive imaging in most patients prior to ICA, the yield for obstructive CAD was <40% in a recent large national study 2. In addition, the prevalence of positive myocardial perfusion imaging studies, has decreased significantly over the last two decades, from 40% to 10% in a recent report 3. Ruling out obstructive CAD in symptomatic women is particularly problematic as currently used diagnostic tests, such as EKG, myocardial perfusion imaging (MPI), and echocardiography, perform less well in women than in men 4. The Agency for Healthcare Research and Quality highlighted the need for a better diagnostic test for women in a recent report by stating that physicians evaluating women at low to intermediate risk of CAD may want to rule-out disease with a non-invasive test with a high negative predictive value 5. In addition, despite the common use of stress testing prior to coronary angiography, practice utilization varies significantly across different regions 6.

## Test Description

When a clinician suspects obstructive CAD as a cause of patient symptoms, a standard venous blood draw is performed into a PAXgene^TM ^RNA preservation tube (Pre-analytix, Valencia, CA). The sample and accompanying test requisition form are sent under temperature controlled conditions to the CLIA and College of American Pathologist’s certified CardioDx laboratory. The sample is then accessioned, and RNA purification, cDNA synthesis, and quantitative real-time polymerase chain reaction (qRT-PCR) are performed. Test results are calculated based on the age and sex of the patient and the expression levels of the 23 genes in the Corus CAD algorithm and reported on a 1-40 scale 7. The test algorithm differs between men and women based on age-dependent risk, specific genes, and relative gene weighting 7. Increasing score is associated with increasing likelihood of obstructive CAD and increasing disease burden. A pre-specified threshold of ≤15 has been prospectively evaluated as a low risk boundary. Approximately 95% of samples received result in a valid test result and of more than 40,000 patients evaluated since launch, 47% have scores below the pre-specified threshold of ≤15.

## Public Health Importance/Prevalence

The evaluation of undiagnosed stable but symptomatic chest pain is associated with as many as 2% of all office visits or 2-3 million visits to primary care outpatient clinics each year in the United States 8 ^,^ 9 ^,^ 10. Patients with symptoms suggestive of CAD undergo extensive non-invasive and invasive testing to exclude the presence of CAD as the cause and, in the process are exposed to risks of iatrogenic side effects such as those from ionizing radiation and contrast dye. The annual U.S. cost of non-invasive imaging tests used in the cardiac work-up of stable symptomatic patients is approximately $7 billion 11 ^,^ 12. Despite the significant resources expended only 10% of patients presenting to primary care with chest pain are ultimately diagnosed with stable obstructive CAD 8.

**Recommendations by independent groups.** As part of the Clinical Laboratory Improvement Act (CLIA) licensure process, the analytical and clinical validation data for the Corus CAD test were independently evaluated by reviewers from the California and New York Departments of Publish Health. In both cases these reviews resulted in positive recommendations and the CardioDx laboratory is licensed in all 50 states. In addition, as of April, 2013, the CardioDx laboratory is now accredited by the College of American Pathologists (CAP).

**Guidelines.** A recent policy statement from the American College of Cardiology and American Heart Association discussed the role of genetics and cardiovascular disease treatment and diagnosis but did not address gene expression as is measured in the Corus CAD Test 13. An earlier scientific statement suggested: “Gene expression profiling has potential application in clinical practice once specific molecular and clinically meaningful CVD signatures are developed” 14.

**Recent Independent Review Articles**. A number of recent independent review articles have described the scientific work underpinning the Corus CAD test 15 ^,^ 16. A very recent review article in Nature Reviews Cardiology was solely focused on Corus CAD 17.

## Evidence Overview

**Analytical Validity**

A large study utilizing more than 800 whole blood control samples was performed to assess the intra and inter-batch variability and inherent reproducibility of the Corus CAD test in the CardioDx commercial laboratory as a function of time, reagent batches, operators, and equipment 18. A total of 11 variables were assessed for their contribution to inter-batch variability, including four individual steps in the process (RNA purification, cDNA synthesis, sample addition, and qRT-PCR) across multiple operator, equipment, and reagent lots. Intra-batch variability estimated from 132 samples was 0.092 Cp units, dominated by inherent PCR stochastic variance, and represented approximately 70% of overall variance. Inter-batch variability was estimated across 895 samples over a two-year time frame; the largest sources of variances were reagent lots, and the overall variance was 0.11 Cp units. A comparison of overall process variability to biological dynamic range across 21,000 clinical samples showed that the biological variability was more than 10 times the process variability, demonstrating that the signal to noise was excellent. Overall process variance standard deviation corresponded to 1 unit on the Corus CAD 1-40 scale, corresponding to a clinically insignificant 1.7% change in obstructive CAD likelihood.

**Clinical Validity**

Two prospective multi-center trials evaluated the performance of the Corus CAD test across populations of different disease prevalence. The PREDICT trial evaluated test performance in a patient population (N=526) referred for invasive coronary angiography, the gold standard for obstructive disease evaluation 19. Disease prevalence was 37%, as measured by core laboratory quantitative coronary angiography, and very similar to that observed in a very large registry study 2. A gender specific analysis of the PREDICT results showed the obstructive CAD prevalence in women was only 22%, indicating a need for better non-invasive diagnostic tools specifically in women 20.

The COMPASS study evaluated test performance in symptomatic patients (N=431) referred for myocardial perfusion imaging, a procedure used prior to angiography 21. The gold standard was a combination of either invasive angiography and CT-angiography, both determined in core laboratories, so that all patients, independent of their MPI results, had gold standard data on their coronary anatomy. Obstructive CAD prevalence was only 15%, lower than seen in PREDICT. Positive MPI scans were seen in 11% of patients, very similar to that seen in a recent study reporting MPI positivity over the last two decades of 10% 3. Results of the two Corus CAD validation studies representing 58 centers in the US were very consistent.

- The primary endpoint of the area under the receiver-operating characteristics curve (AUC for ROC) analysis for discriminating patients with and without obstructive CAD (50% stenosis by quantitative angiography or core-lab CT-angiography) yielded AUCs of 0.70 and 0.79, for PREDICT and COMPASS, respectively (p<0.001 in both cases).

- In both studies Corus CAD demonstrated excellent sensitivity (85 and 89%) and moderate specificity (43 and 52%), respectively, at a threshold of ≤15 which was derived from the PREDICT study and pre-specified for the COMPASS study

- Corus CAD showed high negative predictive values of 83 and 96%, respectively, in the PREDICT and COMPASS studies, consistent with the differences in obstructive CAD prevalence.

- In the subset of PREDICT patients who had MPI and in the entire COMPASS study Corus CAD showed superior diagnostic performance to MPI driven by much greater sensitivity and diagnostic accuracy (ROC curve AUC). In the COMPASS study the AUCs were 0.79, 0.59, and 0.63 for Corus CAD, site-read, and core-lab read MPI, respectively.

- In both studies increasing Corus CAD score was significantly associated with increasing maximum percent stenosis.

- In both studies clinical follow-up for subsequent revascularization and major adverse cardiovascular events was performed. In PREDICT this was for 1 year post-index catheterization and showed a very significant association of Corus CAD score and the composite revascularization and event endpoint 22. In the COMPASS trial 6 month follow-up also showed significantly fewer revascularization and events with low (≤15) Corus CAD scores 21.

**Clinical Utility**

Three studies of the clinical utility of Corus CAD have been reported: a multi-center retrospective chart review in primary care, a prospective single center study of change in behavior in cardiology, and a prospective multi-center change in behavior study in primary care.

Retrospective Chart Review in Primary Care

To document the impact of Corus CAD in real-world primary care practice, a retrospective chart review study was completed in four primary care practices currently using Corus CAD, located in Arizona, Georgia, Louisiana, and North Carolina 23. A total of 317 patients who presented to four primary care physician sites with signs and symptoms suggestive of obstructive CAD and underwent Corus CAD testing from January 2011 to September 2011 were determined to be evaluable by medical records review and were included in this retrospective study. The objective of this study was to determine if there was a relationship between Corus CAD score and referral decision to the cardiologist: specifically, if patients with low Corus CAD scores were less likely to be referred to a cardiologist than patients with non-low scores.

In this study, 41% (129/317) of the Corus CAD patients had low scores (≤15), a rate consistent with the broader commercial population receiving the test and the COMPASS clinical trial population. Based upon physician self-reported referral rates, the expected referral rate to cardiology was 56.5%. The data show that the average referral rate to a cardiologist following Corus CAD testing was reduced to 30% (p<0.001). In addition, the referral rate was just 9% (12/129) in the Corus CAD low scoring patient population. In multivariate analysis, after controlling for age, gender, type of symptoms, and practice site, patients with low Corus CAD scores had a relative reduction in referral likelihood of 73% (p=0.01).

IMPACT-Cardiology

The IMPACT-Cardiology (Investigation of a Molecular Personalized Coronary Gene Expression Test on Cardiology Practice Pattern)(IMPACT-CARD) trial sought to assess the impact of Corus CAD use on clinical decision-making during the assessment of stable chest pain patients in the cardiology setting. The study included a prospective cohort of 83 patients eligible for analysis. These patients were referred to six cardiologists for evaluation of suspected CAD in the Vanderbilt University health care system and were matched by clinical factors to 83 patients in a historical cohort 24. The IMPACT-Cardiology protocol was designed to evaluate and compare the cardiologists’ diagnostic strategies before and after receiving the Corus CAD results for their patients. Clinicians performed a pre-Corus CAD assessment of patients’ CAD probability and noted their preliminary management decision (no intervention/medical management, referral for non-invasive imaging, or referral for invasive angiography). This pre-Corus CAD assessment was compared to physicians’ assessment of CAD probability after seeing the Corus CAD result (post-Corus CAD assessment) and determining a final management decision.

In this study following communication of Corus CAD results, a change in diagnostic testing (e.g. myocardial perfusion imaging, CTA and cardiac catheterization) was noted in 48 patients [58%, 95% CI (46%, 69%)]. More patients had a decreased versus increased level of testing (n=32 (39%) vs n=16 (19%), p=0.03). In particular, 91% (29 of 32) of patients with decreased testing had low Corus CAD (≤ 15), while 100% (16 of 16) of patients with increased testing had elevated Corus CAD (p<0.001). The most common change was among patients considered for referral to non-invasive imaging or invasive angiography prior to the Corus CAD test who were then referred to either no intervention or medical management after receiving a low Corus CAD score. Furthermore, none of the patients with low scores (≤15) saw an increase in testing.

The IMPACT-CARD trial demonstrated that among patients with a low Corus CAD score, the management decisions of cardiologists change, leading to a decrease in non-invasive cardiac imaging and invasive angiography.

IMPACT-PCP

The IMPACT-PCP (Investigation of a Molecular Personalized Coronary Gene Expression Test on Primary Care Practice Pattern) trial assessed the impact of Corus CAD use on clinical decision-making around the assessment of patients with symptoms of obstructive CAD in the primary care setting. The study included a prospective cohort of 251 patients, eligible for analysis, assessed by 8 community based practitioners at four sites. Clinicians performed a pre-Corus CAD assessment of patients’ CAD probability and noted their preliminary management decisions (no intervention/medical management, referral for non-invasive imaging, or referral for invasive angiography). This pre-Corus CAD assessment was compared to the clinician’s assessment of CAD probability after seeing the Corus CAD results (post-Corus CAD assessment) and determining a final management decision.

In this study, a change in diagnostic testing (e.g. myocardial perfusion imaging, CTA and cardiac catheterization) was noted in 145 patients following Corus CAD testing (58% observed vs 10% expected change, p<0.001). More patients had decreased (n=93, 37%) versus increased (n=52, 21%) intensity of testing (p<0.001). In particular, among the 127 low score Corus CAD patients (51% of study patients), 60% (76/127) had decreased testing, and only 2% (3/127) had increased testing. After more than 30 days of follow-up of 247 (98%) patients, there has been one MACE event (hemorrhagic stroke in a low score Corus CAD patient) reported.

In summary, Corus CAD was associated with a statistically significant and clinically relevant change in clinical decision-making among patients evaluated for suspected symptomatic CAD. In addition, the utilization of Corus CAD showed clinical utility above and beyond conventional decision-making by optimizing the patient’s diagnostic evaluation, particularly around the reduction in the intensity of diagnostic testing among low Corus CAD patients.

**Systematic Evidence Reviews**

Palmetto Government Benefits Administrators (Palmetto, GBA), the CMS Medicare Administrative Contractor with oversight for Corus CAD, has published its assessment of the test. This review determined that the test meets standards for analytical and clinical validity, and clinical utility and is a reasonable and necessary Medicare benefit, effective January 1, 2012 25.

Overall Analysis of Evidence. The Evaluation of Genomic Applications in Practice and Prevention (EGAPP) working group has published a framework for evaluation of evidence of genomic testing, comprising analytical and clinical validity and clinical utility 26. For Corus CAD with respect to analytic validity, an extensive study demonstrating very good score reproducibility and the ability to result >95% of samples, suggests a Level 1 evidence category in our judgement, for the performance of the test in the CardioDx clinical laboratory. For clinical validity it is our assessment that the two prospective multi-center trials with core laboratory definition of disease status, representing almost 1000 patients, also correspond to Level 1 evidence according to EGAPP criteria 26. The clinical utility data from the IMPACT and retrospective chart review studies are of relatively small size and limited follow-up suggesting a level 2-3 evidence determination.

## Limitations

Although the results of the evaluation of the Corus CAD test are very promising, its results should be interpreted carefully as patients with diabetes mellitus and chronic inflammatory or autoimmune disorders were excluded from test development and validation. Furthermore, this test was derived and tested in predominantly Caucasian patient populations. Given the known variations in the prevalence of CAD in different ethnic/racial backgrounds 27, results of this test in non-Caucasian populations should be interpreted with caution.

## Conclusions

The Corus CAD test has been extensively evaluated since it was first derived, including with two prospective multi-center trials. Given the scope of the deleterious effects of CAD and the considerable costs involved in diagnosing obstructive CAD, a blood test that can help in this determination is certainly valuable. The Corus CAD test promises to have an important role in this regard particularly if it continues to perform this well in larger, more diverse cohorts.
